# External ventricular drainage management in pediatrics: a simulated scenario for rapid-cycle deliberate practice

**DOI:** 10.1590/0034-7167-2024-0253

**Published:** 2026-09-28

**Authors:** Ruana Luiz Ferreira da Silva, Ana Railka de Souza Oliveira-Kumakura

**Affiliations:** I Universidade Estadual de Campinas. Campinas, São Paulo, Brazil; II Université Paris Cité. Paris, France

**Keywords:** Simulation Training, Pediatric Nursing, Nursing Care, Cerebrospinal Fluid Shunts, High Fidelity Simulation Training., Entrenamiento Simulado, Enfermería Pediátrica, Atención de Enfermería, Derivaciones del Líquido Cefalorraquídeo, Entrenamiento con Simulación de Alta Fidelidad.

## Abstract

**Objectives:**

to construct, assess, and test a simulated scenario using rapid-cycle deliberate practice for external ventricular drainage management in pediatric patients.

**Methods:**

a methodological study, with the construction of a simulated scenario based on the concept of simulated scenario engineering and the NLN Jeffries Simulation Theory. The scenario was assessed by reviewers, who calculated the Modified Kappa Coefficient and Content Validity Index, and then tested it with nursing students.

**Results:**

a scenario was constructed based on updated evidence. Two rounds were conducted with seven evaluators who assessed all elements of the scenario as appropriate, according to adopted references. In the test with the target audience, no adjustments were necessary.

**Conclusions:**

the evidence-based simulated scenario on external ventricular drainage management in pediatrics received an excellent assessment from the evaluators and the target audience during testing. The use of rapid-cycle deliberate practice proved promising for teaching and learning the subject.

## RESUMEN

**Objetivos:**

construir, evaluar y probar un escenario simulado mediante práctica deliberada en ciclos rápidos para el manejo del drenaje ventricular externo en pacientes pediátricos.

**Métodos:**

estudio metodológico, con la construcción de un escenario simulado basado en el concepto de ingeniería de escenarios simulados y la *NLN Jeffries Simulation Theory*. El escenario fue evaluado por revisores, quienes calcularon el Coeficiente Kappa Modificado y el Índice de Validez de Contenido, y lo probaron con estudiantes de enfermería.

**Resultados:**

se construyó un escenario basado en evidencia actualizada. Se realizaron dos rondas con siete revisores, quienes evaluaron todos los elementos del escenario como adecuados, según las referencias adoptadas. En la prueba con el público objetivo, no fue necesario realizar ajustes.

**Conclusiones:**

el escenario simulado basado en la evidencia sobre el manejo del drenaje ventricular externo en pediatría recibió excelentes evaluaciones de los evaluadores y del público objetivo durante la prueba. El uso de la práctica deliberada en ciclos rápidos resultó prometedor para la enseñanza y el aprendizaje del tema.

## INTRODUCTION

Clinical judgment is a non-technical skill that nurses need to develop due to its importance in nursing care, which is permeated by daily clinical decision-making that requires evidence-based reflection to obtain safe and consistent results. Therefore, since this skill can profoundly impact patient outcomes, it must be exercised in a cohesive, consistent, safe, and appropriate manner to provide effective evidence-based care^(1-3)^.

However, the lack of opportunities for students and nurses to develop and refine their clinical judgment is highlighted in studies that show the need to improve learning experiences^(4)^. Therefore, it is necessary to use educational strategies that allow for repetitive practice in a safe and controlled environment, in order to reinforce the process of developing clinical judgment from the training of future nurses^(5,6)^. This will promote the acquisition and development of general knowledge and skills, contributing to a practice of excellence^(7)^.

From this perspective, clinical simulation, in addition to promoting the improvement of psychomotor technical skills, contributes to the development of non-technical skills by allowing the refinement of critical and reflective thinking, enabling the clear and coherent articulation of clinical judgments that will support appropriate interventions, among other things. The results of using this active methodology are diverse, providing students with confidence during their performance, as well as contributing to the healthcare system by promoting better patient outcomes^(8-10)^.

High-fidelity simulation stands out due to its relevance and for providing progress in nursing students’ knowledge and performance, especially in critical care situations. These results validate the importance and effectiveness of using this methodology in the training process of these professionals^(11)^.

Among clinical simulation strategies, rapid-cycle deliberate practice (RCDP) stands out. It is characterized by short, repetitive cycles of specific tasks, briefly interrupted whenever correction is needed. During these pauses, immediate directive feedback is provided, allowing the participant to adjust their practice in real time until mastery is achieved. This process of rapid alternation between practice, feedback, and repetition promotes the continuous improvement of skills^(12-14)^. Previous studies have shown increased self-confidence, satisfaction, and performance among participants when RCDP is used in complex activities^(15)^.

Unlike traditional simulation, where feedback only occurs after the scenario ends (post-event debriefing), in RCDP, feedback is immediate and integrated into the practice session, allowing for quick corrections and immediate consolidation of skills. Previous studies have demonstrated greater effectiveness of RCDP in improving participant performance compared to approaches that offer feedback after the event^(16)^. Additionally, other studies have highlighted the positive effects of RCDP in increasing knowledge acquisition about sepsis in pediatrics, improving the execution of complex medical procedures, and strengthening confidence in pediatric trauma care^(17-19)^.

From this perspective, nursing care, in the context of the proper management of external ventricular drainage (EVD), is fundamental to reducing the risk of complications related to this condition. Therefore, using a teaching methodology that enables participants to immediately perform procedures requiring skills for the proper and safe handling of the EVD system is of utmost importance^(20)^.

Considering the importance of meticulous care regarding the proper management of EVD, using an active teaching methodology that provides participants with the opportunity to hone specific technical skills and improve decision-making in a safe and controlled environment contributes to preparing future professionals to confidently and competently handle real-life situations, in order to promote safe and effective care.

## OBJECTIVES

To construct, validate, and test a simulated scenario using RCDP for EVD management in pediatric patients.

## METHODS

### Ethical aspects

The study was approved by the *Universidade Estadual de Campinas* Research Ethics Committee. All participants involved in the study signed an online (evaluators) and written (nursing students) a consent form.

### Study design, period, and location

This is a methodological study, with a quantitative approach, following the Reporting guidelines for healthcare simulation research: extensions to the CONSORT and STROBE statements recommendations^(21)^, understanding the stages of constructing a simulated scenario using RCDP, which are: 1) construction of a simulated scenario based on the concept of simulated scenario engineering^(22)^ and on the NLN Jeffries Simulation Theory^(23)^; 2) assessment of a scenario by reviewers with experience in the areas of interest of the study, found through a search on the *Lattes* platform; and 3) testing of a simulated scenario with the target audience.

The study was conducted at a public university located in the state of São Paulo, Brazil, and took place from May to September 2023.

### Population or sample; inclusion and exclusion criteria

Stage 2, assessment of a scenario constructed by reviewers, was conducted entirely online. Reviewers were selected by convenience through searches of curricula on the *Lattes* platform via the Brazilian National Council for Scientific and Technological Development. Participants were selected who worked or had worked and possessed knowledge in the areas of interest involving the care of pediatric patients using EVDs and/or clinical simulation. The search prioritized national curricula with publications in the areas of interest and/or *lato sensu* or *stricto sensu* specializations on related topics, as well as available and up-to-date email addresses. Efforts were made to ensure at least one participant from each region of the country, aiming for a heterogeneous sample, considering geographical diversity, potential variations in experience, and practices in the investigated areas. Thus, 17 nurses were selected and received an invitation letter via email with a link to access the electronic form (Google Forms^®^), which included the Informed Consent Form (ICF) for participation in the study and a simulated scenario for assessment. The form was available between May 19 and June 28, 2023, and was completed by seven participants. Exclusion criteria included the absence of publications and/or experience in the areas of interest, as well as duplicate responses.

In stage 3, testing of a simulated scenario, nursing students who had already taken the course on the fundamental aspects of nursing care were invited by email. Only five students from the same academic year agreed to participate. After confirmation of participation, a practical class was scheduled, in which students had initial contact with the EVD system devices and then the simulated scenario began. The limited number of participants resulted from students’ availability and the selection criteria applied. Absence from the practical class was adopted as an exclusion criterion.

### Study protocol

The first stage involved constructing a simulated scenario based on the recommendations proposed by the NLN Jeffries Simulation Theory^(23)^ theoretical model and the engineering of simulated scenarios. The first describes the following components: context; history; participant; facilitator; design; educational practices; simulation experience; and expected results. The second presents the methodological framework, which includes the preparation of the 15 items of the simulated station order, which constitutes the complete script of the simulated scenario, composed of: scenario instructions and student tasks; guidelines for the evaluator; list of materials and equipment; map of the layout of furniture and human resources within the physical environment of the simulated station; script of the simulated patient (if it is a scenic simulation); evaluator decision flowchart; and standardized assessment instrument (checklist)^(22,23)^.

For producing the clinical case, two international guidelines were used, namely Management of Pediatric Severe Traumatic Brain Injury: 2019 Consensus and Guidelines-Based Algorithm for First and Second Tier Therapies, Care of the Patient Undergoing Intracranial Pressure Monitoring/External Ventricular Drainage or Lumbar Drainage - AANN Clinical Practice Guideline Series and the national technical opinion COREN-SP 030/202136 on EVD management by nursing professionals^(24-26)^.

In the second stage, the simulated scenario was validated. The evaluators received an invitation letter by email, which contained a link to the Google Forms^®^ electronic form. Upon acceptance and signing the ICF, the form was divided into two parts. The first was the sociodemographic characterization sheet, which contained the evaluators’ sociodemographic, professional, and academic data. The second part contained the components proposed by the NLN Jeffries Simulation Theory, which would be assessed in relation to the scenario. Each item was assessed using a Likert-type scale from 1 (strongly disagree) to 5 (strongly agree) points, followed by space for suggestions, if any. Only one response per email was allowed, and the presence of the sociodemographic sheet helped to exclude duplicate responses, ensuring the validity and uniqueness of the data collected for the study.

The third stage comprised the scenario testing process with the target audience. The simulated scenario was highly realistic, with an actor playing the role of a professional nurse during shift handover. The person assigned to the role, in addition to having attended drama school, furthered their knowledge by participating in a course on using theater to improve the physician-patient relationship. To ensure performance quality, timely training was provided by the scenario facilitator and principal investigator of the study, given her experience as a clinical simulation instructor. The training included reading the script, analyzing the complete clinical case, and rehearsing the scene, ensuring a realistic and didactic representation. To maintain the realism of the scene, the scenario was set up in the Skills and Simulation Laboratory with devices that comprise a Pediatric Intensive Care Unit, with patients’ bed arranged according to the moment’s needs.

### Data analysis

For data analysis, the Modified Kappa Coefficient (KMC), developed for content validity studies, was used to verify agreement among raters. Values greater than or equal to 0.74 can be considered excellent^(27-30)^. The Content Validity Index (CVI), a method widely used in the health field, was also employed. This index measures the proportion of reviewers who agree with each other on a given aspect of the instrument. The CVI assesses relevance/representativeness using a Likert-type scale with scores ranging from 1 (not relevant or not representative) to 4 (relevant or representative item). Authors indicate that, in the case of assessments carried out by six or more reviewers, a rate above 0.78 is recommended; however, it is noteworthy that the recommended values for verifying the validity of new instruments should be at least 0.90^(31,32)^.

The data collected from the reviewers via the Google Forms^®^ electronic form were organized into a Microsoft Excel^®^ spreadsheet. Subsequently, the MKC and CVI were calculated. For items that obtained a MKC < 0.74 and CVI < 0.90, the items were reformulated according to the suggestions made by reviewers, and the edited material was sent for further assessment.

## RESULTS

### Scenario construction

The constructed scenario was based on literature focusing on the nursing care necessary for the proper management of the EVD drainage system. The scenario development process used RCDP as the chosen simulation type. The scenario consisted of three sequential scenes, which developed according to the clinical case that underpinned its construction. Each scene consisted of a task to be performed by nursing students providing care to pediatric patients using EVD. The necessary care encompassed the proper handling and handling of patients undergoing EVD.

In the final stage, the NLN Jeffries Simulation Theory^(23)^ theoretical model was used to complement the scenario construction with the following stages: pre-briefing, a moment in which the simulated environment and all the resources available in the scene are presented, as well as its operation; briefing, a moment in which information about the clinical case is presented, a simulated scenario that consists of defining the objectives, solving the problems and estimated time for scene development; debriefing, in which the PEARLS health debriefing tool^(33)^ was used; and expected results, which included aspects of cognitive knowledge, clinical judgment and practical skill.

### Content assessment by reviewers

In the assessment phase of the simulated scenario by the evaluators, 17 nurses who met the study’s inclusion criteria were initially invited; however, only seven accepted the invitation and returned within the proposed time period. In the first round of assessment, all items obtained a CVI and MKC greater than 0.86 and 0.85, except for the “scenario content facilitates critical thinking” item, which obtained a CVI of 0.71 and a MKC of 0.66. Therefore, the evaluators’ suggestions were analyzed and accepted in order to improve the constructed simulated scenario content. A second round of assessment was conducted, following the same format as the first, obtaining a CVI and MKC score of 1.00. Suggestions were made to add the process of systematizing nursing care in patients’ medical record to the scenario clues and to address this topic in the debriefing. This suggestion was promptly incorporated into the scenario, as it was considered an important criterion.

The evaluators were mostly female; two evaluators held specialist titles; three had graduate degrees (master’s or doctoral level); and two had postdoctoral degrees. Four evaluators provided care to pediatric patients using EVDs, and all had experience with clinical simulation for a minimum of two and a maximum of eight years. Table 1 presents the MKC and CVI values.

**Table 1:** Content Validity Index and Modified Kappa Coefficient values for each of the items assessed in the complete simulated scenario, Campinas, São Paulo, Brazil, 2024

Item assessed	Round 1 CVI MKC	Round 2 CVI MKC
**Structure/presentation**		
Support provided to participants	1.00 1.00	
Target audience		
Educational practice	1.00 1.00	
Scene fidelity	1.00 1.00	
Problem resolution	0.86 0.85	
Clues	0.86 0.85	
**Debriefing**	1.00 1.00	
Expected results	1.00 1.00	
*Preliminary information (pre-briefing)*	1.00 1.00	
Case summary (briefing)	0.86 0.85	
**Script**	1.00 1.00	
Materials and equipment used	0.86 0.85	
Scientifically accurate information	1.00 1.00	
Logical sequence of content	1.00 1.00	
Information covers content about EVD	1.00 1.00	
Appropriate script for the target audience	0.86 0.85	
Easy-to-understand language	1.00 1.00	
Attractive visual scenario	1.00 1.00	
Data presented in a structured way	1.00 1.00	
**Objectives**		
Content coherence with objectives	0.86 0.85	
Clear and concise learning objectives	1.00 1.00	
Content facilitates critical thinking	0.71 0.66	1.00 1.00
Encourages changes in behavior/attitude	1.00 1.00	
**Relevance**		
Content important for the quality of care provided	1.00 1.00	
Presentation format contributes to learning	0.86 0.85	
Contextual details provide clues based on desired outcomes	0.86 0.85	
Clinical case profile provides sufficient data for clinical judgment	0.86 0.85	
Scenario allows for knowledge transfer related to the topic	0.86 0.85	
Theme portrays key aspects related to clinical practice	1.00 1.00	
Model allows for learning in different contexts	1.00 1.00	
Script proposes knowledge construction	1.00 1.00	
Use by healthcare professionals and/or educators	1.00 1.00	
Scenarios can circulate within the scientific community of the area	1.00 1.00	

*CVI - Content Validity Index; MKC - Modified Kappa Coefficient; EVD - external ventricular drain.*

### Scenario testing

In the scenario test, five sixth-semester nursing students agreed to participate in the study. On the scheduled date, they attended the faculty’s Skills and Simulation Laboratory to participate in a practical class and then in a simulated scenario.

The study’s principal investigator acted as a scenario facilitator, given her experience with clinical simulation and a simulation facilitator course. The stages of good clinical simulation practices proposed by INACSL were followed^(34)^. A professional with experience in clinical simulation was present to observe the facilitator’s execution of the scenario during this testing phase. A checklist, in table form, was used, listing 23 necessary actions to achieve the specific objectives of the scenario (Chart 1), aimed at providing nursing care regarding the proper management of EVD in pediatric patients. This instrument was used to verify the fulfillment or non-fulfillment of each expected action by participants, interrupting the scene when the task was not performed adequately and providing timely feedback, allowing the scene to be repeated until excellence was achieved, as foreseen by the chosen simulation. With the correct execution of the tasks, the scene continued and the scenario unfolded until its conclusion.

**Chart 1:** Brief diagram of the simulated scenario after assessment and testing, Campinas, São Paulo, Brazil, 2023

Scenario title	EVD management in pediatric patients - RCDP
**Purpose**	Facilitating EVD management in pediatric patients
**Loyalty**	High-fidelity simulation
**General objective**	Properly managing EVD in pediatric patients.
**Specific objectives**	Empty the collection bag following the correct sequence of events in order to reduce the risk of infection related to invasive devices; Change the patients’ position, prioritizing patient safety, to reduce the risk of impaired skin integrity related to decreased physical mobility, immobilization, and moisture; Measure the level of the drainage system in relation to patients to monitor the risk of ineffective brain tissue perfusion related to brain injuries (traumatic brain injury).
**Problem-solving**	With moderate complexity, the information relevant to clinical reasoning, such as patients’ general condition, parameters, care provided and care still needed, which will underpin the care provided and the execution of necessary tasks, will be offered during the pre-briefing and debriefing, as well as during the scenario.
**Clues**	a) Drip burette with drained contents inside; b) Collection bag filled to more than ⅔ of its total capacity; c) Nursing prescription without checking the last change of position; d) Hemodynamic instability of the patient upon handling; e) Catheter insertion dressing identified, clean, and dry; f) Nursing care systematization process sheet in patients’ medical record.
**Debriefing**	Emotional stage: how did you feel attending to this patient? Descriptive stage: could you describe patients’ clinical picture? Do you identify any nursing diagnoses? What clinical indicators does this patient present? Evaluative stage: what positive actions did you take? Analytical stage: what would you do differently if you had another opportunity? What types of nursing interventions would you perform, considering the nursing diagnosis(es) you listed? Concluding stage: what did you take away from this experience for your future clinical practice?
**Results**	Cognitive knowledge: measured by the self-assessment scale and knowledge assessment questionnaire regarding EVD management. Clinical judgment: development of the following dimensions: assessment of the risk of hemodynamic instability due to handling of the EVD system; assessment of the risk of pressure injury due to bed restraint; information seeking, prioritization and understanding of data; correlation with the nursing process; calm and confident performance; planned intervention; self-analysis; and commitment to professional development. Practical skill: the initiative and execution of each necessary intervention until excellence is achieved is guaranteed by the type of simulation used.
**Script**	This scenario takes place in a Pediatric Intensive Care Unit. The scene begins with the unit nurse (actor) handing over the shift to nursing students who are interning in the unit and must perform the necessary nursing care according to patients’ needs: a 4-year-old child with severe traumatic brain injury, on the fourth postoperative day, using an EVD, the gold standard for measuring intracranial pressure, with a clean, dry, and valid dressing in place. Development of the scenario divided into scenes, according to each specific objective: Scene 1 - Objective/task: empty the collection bag properly; Scene 2 - Objective/task: safely change the patient’s position; Scene 3 - Objective/task: level the height of the drainage system. The scenario progressed according to participants’ performance, with interruptions occurring if they did not perform the task adequately and follow the set of actions necessary to achieve the objective/task to be performed.

*EVD - external ventricular drainage; RCDP - rapid-cycle deliberate practice.*

At the end of the scenario, all participants gathered for the debriefing, which went through the five indicated stages: preparing the ground to create a safe learning environment; reaction to explore feelings; description to clarify facts; analysis of the moment to explore performance domains; and application to highlight the main lessons.

The simulated scenario was performed simultaneously by all five participants, with a total duration of 15 minutes. During the scenario, three brief pauses were taken to provide immediate directive feedback to participants, allowing for quick adjustments followed by short repetitions of the scene until correct execution in cycles that did not exceed five minutes each, as recommended by RCDP^(35)^.

At the end of the scenario, a post-event debriefing session lasting 18 minutes was conducted to consolidate learning. This format provided a significant improvement in participant performance, ensuring mastery of the skills proposed by the simulated scenario.

No modifications were needed to the setup after testing. The only suggestion was to improve the device that secures the EVD assembly to the IV stand or to replace the stand with a model that makes adjusting its height easier.

## DISCUSSION

This study constructed a simulated scenario using RCDP for EVD management in pediatric patients, planned and structured based on scientific evidence guiding the care of patients using this type of drainage system^(24-26)^.

For the simulated scenario built on EVD management in pediatric patients, the elements proposed by INACSL^(36)^, by NLN Jeffries Simulation Theory and by the concept of simulation scenario engineering^(22)^ were considered, and were appreciated by evaluators in relation to these elements. It was tested with nursing students, the target audience so that it can be used as an educational intervention.

Assessment was conducted by seven reviewers with experience in the areas of interest, clinical simulation, and pediatric patient care using EVDs, and was obtained through the assessment of each criterion that makes up the simulated scenario. The assessed items obtained a CVI and MKC of at least 0.85, considered excellent in terms of the agreement of the assessment of the seven reviewers. The “scenario content facilitates critical thinking” item obtained a CVI of 0.71 and a MKC of 0.66. The suggestions submitted were accepted and subjected to a second assessment, obtaining a CVI and MKC of 1.00. The number of reviewers complies with the minimum of five reviewers, as recommended in the literature for the validity stage^(31,32)^.

The simulated scenario testing process is important and recommended by INACSL guidelines so that, if any, errors or mistakes that may occur during the scenario’s execution can be identified. For this reason, it is necessary that participants in this stage be part of the target audience, as indicated in the literature^(27,37,38)^.

EVD management must be carried out in a coherent and systematic manner. This includes planning followed by the implementation of necessary care in order to keep the patient safe until the therapeutic goal desired by the team can be achieved^(39)^.

RCDP, still underutilized in nursing education, is a simulation modality that has seen increased use due to its demonstrated effectiveness in developing essential clinical practice skills^(35,40)^. Its distinguishing feature lies in providing immediate directive feedback during the simulation, allowing participants to repeat the task until it is executed correctly, enhancing skill retention and self-confidence^(12,16,38,41)^.

The literature shows that carefully structured simulated scenarios favor positive results in knowledge acquisition^(42)^, practical skills, theory-practice correlation, confidence, and self-efficacy. Therefore, to ensure this effectiveness, it is important to follow a rigorous methodological path for scenario construction^(43)^. The second aspect is to use a clinical case based on guidelines that provide guidance on best practices regarding the topic presented. The third aspect is to develop clear learning objectives, following best practices in simulation^(44)^. From this perspective, the SMART method stands out^(4)^ for constructing specific, measurable, achievable, realistic and time-bound learning objectives.

During the present study, participants positively highlighted RCDP, especially for receiving feedback immediately after the scene interruption, allowing for quick correction and repetition until excellence was achieved. Although post-event debriefing is a valuable tool in traditional simulation, in RCDP it takes over a complementary function, occurring after the complete conclusion of the scenario, aiming to consolidate and maximize the learning obtained during interruptions. Therefore, it is essential to adapt the use of debriefing to the purpose of deliberate practice, ensuring alignment and methodological clarity.

It is important to remember that this study represents a preliminary assessment of RCDP in the context studied. Further research is needed to consolidate the findings and improve the methods used. Future studies may confirm the potential of RCDP to improve clinical reasoning and decision-making in critical pediatric situations, with a positive impact on care outcomes and patient safety^(45-47)^.

### Study limitations

The present study was limited by the small number of evaluators available to assess the simulated scenario and the small number of students available for testing.

### Contributions to nursing

EVD management involves careful and meticulous actions, based on critical thinking and prior clinical judgment, to ensure that actions are initiated and executed assertively, guaranteeing adequate and safe nursing care for patients. It is expected that this study will be used in the teaching-learning process of nursing students and nursing professionals who must perform this care, adding theoretical and practical knowledge and refining the practical skills of this group, with a view to solidifying this process and enabling adequate, safe, and high-quality practices in the care of pediatric patients using EVDs.

Clinical simulation, with the appropriate choice of simulation type according to the objectives to be achieved, should be increasingly used in educational institutions in order to promote the correlation of theory with practice and provide nursing students with experience in more complex situations, preparing them for work in real-world practical settings. The choice of RCDP, when possible, is positive because it allows for the training and improvement of practical skills necessary for the proposed care, offering various opportunities for repetition and favoring improved performance.

## CONCLUSIONS

The simulated scenario for managing EVD in pediatric patients was developed based on specific literature on the subject. The construction, validity, and testing process was structured to ensure an effective teaching-learning process, receiving excellent feedback from evaluators and the target audience. RCDP proved to be a promising approach, as it offers immediate feedback and allows for the repetition of tasks until excellence is achieved.

Widely used in healthcare training, clinical simulation offers a safe and controlled environment, allowing students to be exposed to complex cases without causing harm to real patients. In this context, realistic scenarios provide rich experiences, promoting the improvement of technical and non-technical skills, such as communication, teamwork, and decision-making. Applied to pediatric endocarditis management, clinical simulation reinforces its value as an innovative educational tool, contributing significantly to nursing care quality and safety.

## Data Availability

The research data are available in a repository: https://doi.org/10.25824/redu/KXBNDW.
