# Supplementary material for: External ventricular drainage management in pediatrics: a simulated scenario for rapid-cycle deliberate practice
Source: Rev Bras Enferm. 2026 Sep 28;79:e20240253. doi: 10.1590/0034-7167-2024-0253 (PMC13623300; doi:10.1590/0034-7167-2024-0253)
Supplement: Translation

# Translation: Manejo da derivação ventricular externa em pediatria: cenário simulado para prática deliberada em ciclos rápidos

**DOI:** 10.1590/0034-7167-2024-0253pt

**Authors:** Ruana Luiz Ferreira da Silva, Ana Railka de Souza Oliveira-Kumakura

**Affiliations:** I Universidade Estadual de Campinas. Campinas, São Paulo, Brasil; II Université Paris Cité. Paris, França

**Keywords:** Treinamento por Simulação, Enfermagem Pediátrica, Cuidados de Enfermagem, Derivações do Líquido Cefalorraquidiano, Treinamento com Simulação de Alta Fidelidade.

## Abstract

**Objetivos:**

construir, avaliar e testar cenário simulado usando a prática deliberada em ciclos rápidos para manejo da derivação ventricular externa no paciente pediátrico.

**Métodos:**

estudo metodológico, com construção de cenário simulado baseado no conceito de engenharia dos cenários simulados e na *NLN Jeffries Simulation Theory*. O cenário foi apreciado por avaliadores, com cálculo do Coeficiente de Kappa Modificado e do Índice de Validade de Conteúdo, sendo testado com estudantes de enfermagem.

**Resultados:**

construiu-se cenário com base em evidências atualizadas. Realizaram-se duas rodadas com sete avaliadores que apreciaram todos os elementos do cenário adequados, conforme as referências adotadas. No teste com o público-alvo, não houve necessidade de ajustes.

**Conclusões:**

o cenário simulado baseado em evidências sobre o manejo da derivação ventricular externa em pediatria recebeu excelente avaliação dos avaliadores e do público-alvo durante a testagem. O uso da prática deliberada em ciclos rápidos mostrou-se promissor para o ensino-aprendizagem da temática.

## INTRODUÇÃO

O julgamento clínico é uma habilidade não técnica que o enfermeiro precisa desenvolver devido à sua importância para o cuidado de enfermagem, que é permeado de tomadas de decisão clínicas diárias que exigem reflexão pautada em evidências para se obter resultados seguros e coerentes. Logo, como essa habilidade pode impactar profundamente os resultados dos pacientes, ela deve ser realizada de modo coeso, consistente, seguro e adequado, a fim de proporcionar um cuidado eficaz baseado em evidências^(1-3)^.

No entanto, a falta de oportunidades para que estudantes e enfermeiros possam desenvolver e aperfeiçoar o seu julgamento clínico é destacada em estudos que mostram a necessidade de melhorar as experiências de aprendizagem^(4)^. Portanto, é necessária a utilização de estratégias educacionais que permitam a prática repetitiva em um ambiente seguro e controlado, a fim de reforçar o processo de desenvolvimento do julgamento clínico desde a formação dos futuros enfermeiros^(5,6)^. Isso irá favorecer a aquisição e desenvolvimento do conhecimento e de competências gerais, contribuindo para uma prática de excelência^(7)^.

Nessa perspectiva, a simulação clínica, além de favorecer o aprimoramento de habilidades técnicas psicomotoras, contribui com o desenvolvimento de habilidades não técnicas, ao permitir o refinamento do pensamento crítico e reflexivo, possibilita a articulação de forma clara e coerente dos julgamentos clínicos que subsidiarão intervenções adequadas, entre outras. Os resultados da utilização dessa metodologia ativa são diversos, fornecendo segurança aos estudantes durante o seu desempenho, além de contribuir com o sistema de saúde, ao favorecer melhores resultados para os pacientes^(8-10)^.

A simulação de alta fidelidade se sobressai face sua relevância e por proporcionar progresso no conhecimento e desempenho dos estudantes de enfermagem, principalmente em situações de atendimento onde o cenário é de gravidade. Tais resultados legitimam a importância e efetividade do uso dessa metodologia no processo formativo desse profissional^(11)^.

Entre as estratégias de simulação clínica, destaca-se a prática deliberada em ciclos rápidos (PDCR), caracterizada por ciclos curtos e repetitivos de tarefas específicas, que são interrompidos brevemente sempre que há necessidade de correção. Nessas pausas, ocorre a oferta imediata de *feedback* diretivo, permitindo ao participante ajustar a prática, em tempo real, até atingir a maestria. Esse processo de alternância rápida entre prática, *feedback* e repetição favorece o aprimoramento contínuo das habilidades^(12-14)^. Estudos prévios demonstram aumento na autoconfiança, na satisfação e nos desempenhos dos participantes quando utilizada a PDCR em atividades complexas^(15)^.

Ao contrário da simulação tradicional, em que o *feedback* ocorre apenas após o término do cenário (*debriefing* pós-evento), na PDCR, o *feedback* é imediato e integrado ao momento da prática, permitindo correções rápidas e consolidação imediata das habilidades. Estudos prévios têm demonstrado maior efetividade da PDCR em melhorar o desempenho dos participantes em comparação com abordagens que oferecem *feedback* após o evento^(16)^. Adicionalmente, outros estudos destacaram os efeitos positivos da PDCR para aumentar a aquisição de conhecimento sobre sepse em pediatria, aprimorar a execução de procedimentos médicos complexos e fortalecer a confiança no atendimento de trauma pediátrico^(17-19)^.

Nessa perspectiva, o cuidado de enfermagem, diante do manejo adequado da derivação ventricular externa (DVE), é fundamental para reduzir riscos de complicações relacionados a essa situação. Dessa forma, utilizar uma metodologia de ensino que possibilite a ação imediata do participante com a execução de procedimentos que requerem habilidades para uma manipulação adequada e segura do sistema de DVE é de extrema importância^(20)^.

Considerando a importância do cuidado minucioso acerca do manejo adequado da DVE, utilizar uma metodologia de ensino ativa que proporciona aos participantes a oportunidade de lapidar habilidades técnicas específicas e aprimorar a tomada de decisão em ambiente seguro e controlado contribui para o preparo de futuros profissionais para lidar de modo confiante e competente com situações reais, a fim de promover um cuidado seguro e eficaz.

## OBJETIVOS

Construir, validar e testar um cenário simulado com o uso da PDCR para manejo da DVE no paciente pediátrico.

## MÉTODOS

### Aspectos éticos

O estudo foi aprovado pelo Comitê de Ética em Pesquisa da Universidade Estadual de Campinas. Todos os participantes envolvidos no estudo assinaram consentimento de anuência *online* (avaliadores) e escrito (estudantes de enfermagem).

### Desenho, período e local do estudo

Estudo metodológico, de abordagem quantitativa, seguindo as recomendações do *Reporting guidelines for health care simulation research: extensions to the CONSORT and STROBE statements* ^(21)^, compreendendo as etapas de construção de um cenário simulado utilizando a PDCR, sendo elas: 1) construção do cenário simulado baseado no conceito de engenharia dos cenários simulados^(22)^ e na *NLN Jeffries Simulation Theory* ^(23)^; 2) apreciação do cenário por avaliadores com experiência nas áreas de interesse do estudo, encontrados por meio de busca na plataforma *Lattes*; e 3) teste do cenário simulado com o público-alvo.

O estudo foi realizado em universidade pública localizada no estado de São Paulo, Brasil, e aconteceu de maio a setembro de 2023.

### População ou amostra; critérios de inclusão e exclusão

A etapa 2, apreciação do cenário construído por avaliadores, foi realizada integralmente de maneira *online*. Os avaliadores foram selecionados por conveniência por meio de buscas dos currículos na plataforma *Lattes* via Conselho Nacional de Desenvolvimento Científico e Tecnológico. Foram selecionados participantes que atuassem ou tivessem atuado e possuíssem conhecimento nas áreas de interesse que envolvem o cuidado do paciente pediátrico em uso de DVE e/ou simulação clínica. A busca priorizou currículos nacionais com publicações nas áreas de interesse e/ou especializações *lato sensu* ou *stricto sensu* com temática nas áreas relacionadas, além de endereços eletrônicos disponíveis e atualizados. Preocupou-se em garantir pelo menos um convidado de cada região do país, a fim de tentar uma amostra heterogênea, considerando a diversidade geográfica, a potencial variação de experiências e as práticas nas áreas investigadas. Dessa forma, foram elencados 17 enfermeiros, que receberam uma carta convite por *e-mail* com um *link* para acessar o formulário eletrônico (*Google Forms* ^®^), composto pelo Termo de Consentimento Livre e Esclarecido (TCLE), para participação no estudo e pelo cenário simulado construído para apreciação. O formulário ficou disponível entre 19 de maio e 28 de junho de 2023, e foi respondido por sete participantes. O critério de exclusão do estudo abrangeu ausência de publicações e/ou experiência nas áreas de interesse, além de respostas duplicadas.

Na etapa 3, teste do cenário simulado, foram convidados por e-mail estudantes de enfermagem que já haviam cursado a disciplina sobre os aspectos fundamentais do cuidar em enfermagem. Apenas cinco estudantes do mesmo período letivo aceitaram participar. Após a confirmação da participação, foi agendada uma aula prática, na qual os estudantes tiveram contato inicial com os dispositivos do sistema de DVE e, em seguida, iniciou-se o cenário simulado. O número limitado de participantes decorreu da disponibilidade dos estudantes e do critério de seleção aplicado. A ausência na aula prática foi adotada como critério de exclusão.

### Protocolo do estudo

A primeira etapa compreendeu a construção do cenário simulado embasado pelas recomendações propostas pelo modelo teórico da *NLN Jeffries Simulation Theory* ^(23)^ e da engenharia dos cenários simulados. A primeira descreve os seguintes componentes: contexto; histórico; participante; facilitador; *design;* práticas educativas; experiência de simulação; e resultados esperados. A segunda apresenta o referencial metodológico, que contempla a preparação dos 15 itens da encomenda da estação simulada, que se constitui no roteiro completo do cenário simulado, composto por: instruções do cenário e tarefas do estudante; orientações ao avaliador; lista de materiais e equipamentos; mapa de disposição dos móveis e recursos humanos dentro do ambiente físico da estação simulada; *script* do paciente simulado (caso seja simulação cênica); fluxograma de decisão do avaliador; e instrumento padronizado de avaliação (*checklist*)^(22,23)^.

Para a produção do caso clínico, utilizaram-se dois *guidelines* internacionais, sendo eles o *Management of Pediatric Severe Traumatic Brain Injury: 2019 Consensus and Guidelines-Based Algorithm for First and Second Tier Therapies, Care of the Patient Undergoing Intracranial Pressure Monitoring/External Ventricular Drainage or Lumbar Drainage - AANN Clinical Practice Guideline Series* e o parecer técnico nacional COREN-SP Nº 030/202136 sobre o manejo da DVE por profissionais de enfermagem^(24-26)^.

Na segunda etapa, ocorreu a validação do cenário simulado construído. Os avaliadores receberam a carta convite por e-mail, que continha o *link* para o formulário eletrônico *Google Forms* ^®^. Após o aceite e a assinatura do TCLE, o formulário era dividido em duas partes. A primeira era a ficha de caracterização sociodemográfica, que continha dados sociodemográficos, profissionais e acadêmicos dos avaliadores. A segunda parte continha os componentes propostos pela *NLN Jeffries Simulation Theory*, que seriam apreciados em relação ao cenário. Cada item foi avaliado utilizando uma escala tipo Likert de 1 (discordo totalmente) a 5 (concordo totalmente) pontos, seguido por espaço destinado às sugestões, caso houvesse. Era permitido apenas uma única resposta por e-mail, e a presença da ficha sociodemográfica subsidiou a exclusão de respostas duplicadas, garantindo a validade e unicidade dos dados coletados para o estudo.

A terceira etapa compôs o processo de teste de cenário com o público-alvo. O cenário simulado era de alta fidelidade, com participação de ator para desenvolver o papel do profissional enfermeiro na passagem de plantão. A pessoa designada para a atuação, além de ter feito escola de teatro, aperfeiçoou seus conhecimentos participando de uma disciplina cujo tema versava sobre o teatro para o aperfeiçoamento da relação médico-paciente. Para garantir a qualidade da encenação, foi realizado um treinamento oportuno pela facilitadora do cenário e pesquisadora principal do estudo, por ser instrutora em simulação clínica e apresentar experiência na área. O treinamento incluiu a leitura do *script*, análise do caso clínico completo e ensaio da cena, assegurando uma representação realista e didática. A fim de manter o realismo da cena, o cenário foi organizado no Laboratório de Habilidades e Simulação com dispositivos que compõem uma Unidade de Terapia Intensiva Pediátrica, sendo o leito do paciente em questão organizado de acordo com as necessidades do momento.

### Análise de dados

Para a análise de dados, utilizou-se o Coeficiente de Kappa Modificado (CKM), desenvolvido para estudos de validade de conteúdo para verificar a concordância entre os avaliadores. Valores superiores e iguais a 0,74 podem ser considerados excelentes^(27-30)^. Utilizou-se também o Índice de Validade de Conteúdo (IVC), método bastante usado na área da saúde, que mensura a proporção de avaliadores que concordam entre si sobre determinado aspecto do instrumento. O IVC avalia a relevância/representatividade por meio de escala tipo Likert com pontuação de 1 (não relevante ou não representativo) a 4 (item relevante ou representativo). Autores indicam que, no caso de apreciação realizada por seis ou mais avaliadores, recomenda-se uma taxa acima de 0,78; entretanto, destaca-se que os valores recomendados para verificar a validade de novos instrumentos devem ser de no mínimo 0,90^(31,32)^.

Os dados coletados com os avaliadores por meio do formulário eletrônico *Google Forms* ^®^ foram organizados em planilha do *Microsoft Excel* ^®^. Posteriormente, foram calculados o CKM e o IVC. Para os itens que obtiveram CKM < 0,74 e IVC < 0,90, houve a reformulação conforme as sugestões realizadas pelos avaliadores e envio do material editado para nova apreciação.

## RESULTADOS

### Construção do cenário

O cenário construído foi pautado na literatura com foco nos cuidados de enfermagem necessários para o adequado manejo do sistema de drenagem da DVE. O percurso de desenvolvimento da construção do cenário utilizou a PDCR como tipo de simulação escolhido. O cenário foi composto por três cenas sequenciais, que se desenvolveram de acordo com o caso clínico que embasou sua construção. Cada cena foi composta por uma tarefa a ser executada pelo estudante de enfermagem que prestava assistência ao paciente pediátrico em uso da DVE. Os cuidados necessários abrangiam o manejo e a manipulação adequados do paciente submetido à DVE.

Na finalização desta etapa, utilizou-se o modelo teórico da *NLN Jeffries Simulation Theory* ^(23)^ para complementar a construção do cenário com as seguintes etapas: pré-*briefing,* momento no qual são apresentados o ambiente simulado e todos os recursos disponíveis na cena, assim como seu funcionamento; *briefing*, momento em que se apresentam as informações acerca do caso clínico, cenário simulado que consiste na definição dos objetivos, resolução dos problemas e tempo estimado de desenvolvimento das cenas; *debriefing,* no qual foi utilizada a ferramenta de *debriefing* em saúde *PEARLS* ^(33)^; e resultados esperados, o qual contemplou os aspectos de conhecimento cognitivo, julgamento clínico e habilidade prática.

### Avaliação do conteúdo pelos avaliadores

Na etapa de avaliação do cenário simulado pelos avaliadores, foram convidados, inicialmente, 17 enfermeiros que atendiam aos critérios de inclusão do estudo; no entanto, apenas sete aceitaram o convite e retornaram dentro do período de tempo proposto. Na primeira rodada de avaliação, todos itens obtiveram IVC e CKM maior que 0,86 e 0,85, exceto o item “conteúdo do cenário facilita o pensamento crítico”, que obteve IVC de 0,71 e CKM de 0,66. Dessa forma, foram analisadas e acatadas as sugestões dos avaliadores, a fim de aprimorar o conteúdo do cenário simulado construído. Realizou-se uma segunda rodada de avaliação, seguindo o mesmo formato da primeira, obtendo-se um IVC e CKM de 1,00. Houve sugestões para adicionar às pistas do cenário o processo de sistematização da assistência de enfermagem no prontuário do paciente e para abordar esse tema no *debriefing*. Tal sugestão foi prontamente incorporada ao cenário, por ser considerado um critério importante.

Os avaliadores foram em sua maioria do sexo feminino; dois avaliadores apresentavam título de especialista; três possuíam pós-graduação *stricto sensu* nível mestrado ou doutorado; e dois possuíam pós-doutorado. Quatro avaliadores prestaram assistência a pacientes pediátricos em uso de DVE, e todos possuem experiência com simulação clínica por no mínimo dois e no máximo oito anos. Na Tabela 1, estão apresentados os valores de CKM e IVC.

**Tabela 1:** Valores do Índice de Validade de Conteúdo e do Coeficiente de Kappa Modificado para cada um dos itens avaliados no cenário simulado completo, Campinas, São Paulo, Brasil, 2024

Item avaliado	1ª rodada IVC CKM	2ª rodada IVC CKM
**Estrutura/apresentação**		
Suporte providenciado ao participante	1,00 1,00	
Público-alvo	0,86 0,85	
Prática educativa	1,00 1,00	
Fidelidade do cenário	1,00 1,00	
Resolução do problema	0,86 0,85	
Pistas	0,86 0,85	
***Debriefing***	1,00 1,00	
Resultados esperados	1,00 1,00	
Informação prévia (pré-*briefing)*	1,00 1,00	
Sumário do caso (*briefing*)	0,86 0,85	
***Script***	1,00 1,00	
Materiais e equipamento utilizados	0,86 0,85	
Informações cientificamente corretas	1,00 1,00	
Sequência lógica de conteúdo	1,00 1,00	
Informações abrangem o conteúdo sobre DVE	1,00 1,00	
Roteiro apropriado para público-alvo	0,86 0,85	
Linguagem de fácil compreensão	1,00 1,00	
Visual atraente do cenário	1,00 1,00	
Dados apresentados de modo estruturado	1,00 1,00	
**Objetivos**		
Coerência do conteúdo com os objetivos	0,86 0,85	
Objetivos de aprendizagem claros e concisos	1,00 1,00	
Conteúdo facilita pensamento crítico	0,71 0,66	1,00 1,00
Instigam mudanças de comportamento/atitude	1,00 1,00	
**Relevância**		
Conteúdo importantes para a qualidade da assistência prestada	1,00 1,00	
Forma de apresentação contribui para o aprendizado	0,86 0,85	
Detalhes contextuais fornecem pistas com base nos resultados desejados	0,86 0,85	
Perfil do caso clínico fornece dados suficientes para o julgamento clínico	0,86 0,85	
Cenário permite a transferência de conhecimento em relação à temática	0,86 0,85	
Tema retrata aspectos-chave em relação à prática clínica	1,00 1,00	
Modelo permite aprendizado em diferentes contextos	1,00 1,00	
Roteiro propõe a construção do conhecimento	1,00 1,00	
Utilização por profissionais de saúde e/ou educadores	1,00 1,00	
Cenários podem circular no meio científico da área	1,00 1,00	

*IVC - Índice de Validade de Conteúdo; CKM - Coeficiente de Kappa Modificado; DVE - derivação ventricular externa.*

### Testagem do cenário

No teste do cenário, cinco estudantes do sexto período do curso de enfermagem aceitaram e participaram do estudo. Na data agendada, compareceram ao Laboratório de Habilidades e Simulação da faculdade para participar de uma aula prática e, em seguida, de um cenário simulado.

A pesquisadora principal do estudo atuou como facilitadora do cenário, por ter experiência com simulação clínica e curso de facilitador em de simulação. Respeitaram-se as etapas de boas práticas em simulação clínica propostas pela *INACSL* ^(34)^. Contou-se com a presença de um profissional, com experiência em simulação clínica, para observar a execução do cenário pela facilitadora nessa fase de teste. Apoiou-se no *checklist* elaborado, em forma de tabela, listando 23 ações necessárias a serem executadas de forma a alcançar os objetivos específicos do cenário (Quadro 1), visando à execução do cuidado de enfermagem acerca do manejo adequado da DVE no paciente pediátrico. Esse instrumento foi utilizado para verificar o cumprimento ou não de cada ação esperada pelos participantes, interrompendo a cena quando a tarefa não era desempenhada de forma adequada e direcionando um *feedback* oportuno, permitindo a repetição da cena até que se alcançasse a excelência, conforme prevê a simulação escolhida. Com a execução correta das tarefas, a cena se seguia e o cenário continuava a se desenvolver até a sua conclusão.

**Quadro 1:** Desenho resumido do cenário simulado após apreciação dos avaliadores e testagem, Campinas, São Paulo, Brasil, 2023

Título do cenário	O manejo da DVE no paciente pediátrico - PDCR
**Finalidade**	Favorecer o manejo da DVE no paciente pediátrico
**Fidelidade**	Simulação de alta fidelidade
**Objetivo geral**	Manejar de forma adequada a DVE no paciente pediátrico.
**Objetivos específicos**	Esvaziar bolsa coletora seguindo a ordem correta dos eventos, a fim de reduzir o risco de infecção relacionada a dispositivos invasivos; Mudar decúbito do paciente, prezando pela segurança do paciente, para diminuir o risco de integridade da pele prejudicado relacionado à diminuição da mobilidade física, imobilização e umidade; Mensurar o nível do sistema de drenagem em relação ao paciente para monitorar o risco de perfusão de tecido cerebral ineficaz relacionado às lesões cerebrais (traumatismo crânio encefálico).
**Resolução de problemas**	Com complexidade moderada, as informações pertinentes para o raciocínio clínico, como o estado geral do paciente, os parâmetros, os cuidados realizados e os que ainda precisam ser feitos, que embasarão o atendimento com a execução das tarefas necessárias, serão oferecidas durante o pré-*briefing* e o *debriefing*, bem como durante o cenário.
**Pistas**	a) Bureta de gotejamento com conteúdo drenado em seu interior; b) Bolsa coletora preenchida com mais de ⅔ da capacidade total; c) Prescrição de enfermagem sem checar última mudança de decúbito; d) Instabilidade hemodinâmica do paciente à manipulação; e) Curativo de inserção do cateter identificado, limpo e seco; f) Folha do processo de sistematização da assistência de enfermagem no prontuário do paciente.
***Debriefing***	Estágio emocional: como você se sentiu atendendo este paciente? Estágio descritivo: você poderia descrever o quadro clínico do paciente? Você identifica algum diagnóstico de enfermagem? Quais indicadores clínicos esse paciente apresenta? Estágio avaliativo: quais foram as ações positivas que realizou? Estágio analítico: o que você faria de diferente se tivesse outra oportunidade? Quais tipos de intervenções de enfermagem você faria, pensando no(s) diagnóstico(s) de enfermagem que você elencou? Estágio conclusivo: o que você leva de aprendizado desta experiência para sua prática clínica futura?
**Resultados**	Conhecimento cognitivo: mensurado pela escala de autoavaliação e questionário de avaliação do conhecimento sobre o manejo da DVE. Julgamento clínico: desenvolvimento das seguintes dimensões: avaliação do risco de instabilidade hemodinâmica devido à manipulação do sistema da DVE; avaliação do risco de lesão por pressão devido à restrição no leito; busca por informações, priorização e compreensão dos dados; correlação com o processo de enfermagem; atuação calma e confiante; intervenção planejada; autoanálise; e comprometimento com o aperfeiçoamento profissional. Habilidade prática: iniciativa e execução de cada intervenção necessária até o alcance da excelência, garantido pelo tipo de simulação utilizada.
***Script***	Este cenário ocorre em uma Unidade de Terapia Intensiva Pediátrica. O cenário começa com o enfermeiro do setor (ator) passando o plantão para os estudantes de enfermagem que estão estagiando na unidade e deverão desempenhar os cuidados de enfermagem necessários de acordo com as demandas do paciente: uma criança de 4 anos de idade, com traumatismo cranioencefálico grave, no quarto dia de pós-operatório, em uso de DVE, padrão-ouro para mensuração da pressão intracraniana, com curativo em inserção limpo, seco e dentro da validade. Desenvolvimento do cenário dividido em cenas, de acordo com cada objetivo específico: Cena 1 - Objetivo/tarefa: esvaziamento da bolsa coletora de forma adequada; Cena 2 - Objetivo/tarefa: mudança de decúbito do paciente de forma segura; Cena 3 - Objetivo/tarefa: nivelamento da altura do sistema de drenagem. O cenário seguia de acordo com o desempenho dos participantes, sendo interrompidos caso não realizassem a tarefa de forma adequada e seguindo o conjunto de ações necessárias para o desempenho de objetivo/tarefa a ser realizada.

*DVE - derivação ventricular externa; PDCR - prática deliberada em ciclos rápidos.*

Ao término do cenário, todos os participantes se reuniram para o *debriefing*, que passou pelos cinco passos indicados: preparação do terreno para criar um ambiente seguro de aprendizado; reação para explorar os sentimentos; descrição para esclarecer fatos; análise do momento para explorar os domínios de desempenho; e aplicação para destacar as lições principais.

O cenário simulado foi realizado simultaneamente pelos cinco participantes, com duração total de 15 minutos. Durante o cenário, foram realizadas três pausas breves para oferecer *feedback* diretivo imediato aos participantes, permitindo ajustes rápidos seguidos por repetições curtas da cena até a execução correta em ciclos que não ultrapassaram cinco minutos cada, conforme recomenda a PDCR^(35)^.

Ao final do cenário, foi conduzido um *debriefing* pós-evento, com duração de 18 minutos, para consolidar o aprendizado. Esse formato proporcionou uma melhora significativa na performance dos participantes, assegurando o domínio das habilidades propostas pelo cenário simulado.

Não houve modificações a serem feitas no cenário após a testagem. A única sugestão foi melhorar o dispositivo que fixa o conjunto da DVE no suporte de soro ou trocar o suporte para um modelo que torne a manipulação de sua altura mais fácil.

## DISCUSSÃO

O presente estudo construiu um cenário simulado com o uso da PDCR para o manejo da DVE no paciente pediátrico, planejado e estruturado com base em evidências científicas que orientam o cuidado do paciente que faz uso desse tipo de sistema de drenagem^(24-26)^.

Para o cenário simulado construído sobre o manejo da DVE no paciente pediátrico, consideraram-se os elementos propostos pela INACSL^(36)^, pela *NLN Jeffries Simulation Theory* e pelo conceito de engenharia dos cenários simulados^(22)^, sendo apreciado por avaliadores em relação a esses elementos. Foi testado com estudantes de enfermagem, público-alvo, para que possa ser utilizado como intervenção educativa.

A avaliação foi realizada por sete avaliadores com experiência nas áreas de interesse, simulação clínica e cuidados ao paciente pediátrico em uso de DVE, sendo obtida por meio da avaliação de cada critério que compõe o cenário simulado. Os itens avaliados obtiveram IVC e CKM de, no mínimo, 0,85, considerados excelentes no que se refere à concordância da avaliação dos sete avaliadores. O item “conteúdo do cenário facilita o pensamento crítico” obteve IVC de 0,71 e CKM de 0,66. As sugestões enviadas foram acolhidas e submetidas a uma segunda avaliação, obtendo IVC e CKM de 1,00. O número de avaliadores obedece ao mínimo de cinco avaliadores, conforme as recomendações da literatura para a etapa de validação^(31,32)^.

O processo de testagem do cenário simulado é importante e recomendado pelas diretrizes da INACSL para que, se houver, seja possível identificar erros ou equívocos que podem ocorrer durante a execução do cenário. Por esse motivo, é necessário que o participante desta etapa faça parte do público-alvo, conforme a literatura indica^(27,37,38)^.

O manejo da DVE deve ser feito de forma coerente e sistematizada. Isso inclui o planejamento seguido da implementação de cuidados necessários, a fim de manter o paciente seguro, até que seja possível alcançar a meta terapêutica almejada pela equipe^(39)^.

A PDCR, ainda pouco utilizada no ensino de enfermagem, é uma modalidade de simulação que tem ampliado seu uso devido à sua eficácia demonstrada no desenvolvimento de competências essenciais à prática clínica^(35,40)^. Seu diferencial consiste no fornecimento imediato de *feedback* diretivo durante a simulação, permitindo ao participante repetir a tarefa até a execução correta, potencializando a retenção de habilidades e a autoconfiança^(12,16,38,41)^.

A literatura evidencia que cenários simulados cuidadosamente estruturados favorecem resultados positivos em aquisição de conhecimento^(42)^, habilidades práticas, correlação teoria-prática, confiança e autoeficácia. Dessa maneira, para garantir essa efetividade, é importante seguir percurso metodológico rigoroso para construção de cenário^(43)^. O segundo aspecto é utilizar um caso clínico pautado em diretrizes que orientem as boas práticas diante do tema exposto. O terceiro aspecto é elaborar objetivos de aprendizagem claros, seguindo as boas práticas em simulação^(44)^. Nesta perspectiva, sobressai o método *SMART* ^(4)^ para a construção de objetivos de aprendizagem específicos, mensuráveis, alcançáveis, realistas e com prazo definido.

Durante o presente estudo, os participantes destacaram positivamente a PDCR, especialmente por receberem *feedback* imediatamente após a interrupção da cena, permitindo correção rápida e repetição até alcançar a excelência. Embora o *debriefing* pós-evento seja uma ferramenta valiosa na simulação tradicional, na PDCR, ele assume uma função complementar, ocorrendo após a conclusão total do cenário, visando consolidar e maximizar o aprendizado obtido durante as interrupções. Por isso, é essencial adaptar o uso do *debriefing* ao propósito da prática deliberada, garantindo alinhamento e clareza metodológica.

Vale lembrar que este estudo representa uma avaliação preliminar da PDCR no contexto estudado. Novas investigações são necessárias para consolidar os achados e aprimorar os métodos utilizados. Estudos futuros podem confirmar o potencial da PDCR em aprimorar o raciocínio clínico e a tomada de decisão em situações pediátricas críticas, com impacto positivo nos resultados assistenciais e na segurança do paciente^(45-47)^.

### Limitações do estudo

O presente estudo teve como limitações o reduzido número de avaliadores para a apreciação do cenário simulado e de estudantes para a testagem.

### Contribuições para a área da enfermagem

O manejo da DVE é composto por ações criteriosas e minuciosas, embasadas em pensamento crítico e julgamento clínico prévio, para que as ações sejam iniciadas e executadas de modo assertivo, garantindo um cuidado de enfermagem adequado e seguro para o paciente. Espera-se que o presente estudo seja utilizado no processo de ensino-aprendizagem de estudantes de enfermagem e de profissionais de enfermagem que devem realizar este cuidado, agregando conhecimento teórico-prático e lapidando habilidades práticas deste público, com vistas a solidificar esse processo e viabilizar práticas adequadas, seguras e de qualidade no cuidado do paciente pediátrico em uso de DVE.

A simulação clínica, com escolha adequada do tipo de simulação diante dos objetivos a serem alcançados, deve ser cada vez mais utilizada em instituições de ensino, a fim de promover correlação da teoria com a prática e oportunizar a vivência de estudantes de enfermagem em situações de maior complexidade, preparando-o para atuação no cenário prático real. A escolha da PDCR, quando possível, é positiva, pois permite treinar e aperfeiçoar as habilidades práticas necessárias ao cuidado proposto, por oferecer diversas oportunidades de repetição, favorecendo uma melhora no desempenho.

## CONCLUSÕES

O cenário simulado para o manejo da DVE no paciente pediátrico foi desenvolvido com base em literatura específica no tema. O processo de construção, validação e testagem foi estruturado para assegurar um processo de ensino-aprendizagem eficaz, recebendo excelente apreciação dos avaliadores e público-alvo. A PDCR se mostrou uma abordagem promissora, pois oferece *feedback* imediato e permite a repetição das tarefas até alcançar a excelência.

Amplamente utilizada na formação em saúde, a simulação clínica propõe um ambiente seguro e controlado, permitindo que estudantes sejam expostos a casos complexos sem causar danos a pacientes reais. Nesse contexto, cenários realistas proporcionam experiências ricas, promovendo o aprimoramento de habilidades técnicas e não técnicas, como comunicação, trabalho em equipe e tomada de decisão. Aplicada ao manejo da DVE pediátrica, a PDCR reforça seu valor como ferramenta educacional inovadora, contribuindo significativamente para a qualidade e segurança do cuidado em enfermagem.

## Data Availability

Os dados de pesquisa estão disponíveis em repositório: https://doi.org/10.25824/redu/KXBNDW.
